# Transcatheter or surgical aortic valve implantation: 10-year outcomes of the NOTION trial

**DOI:** 10.1093/eurheartj/ehae043

**Published:** 2024-02-07

**Authors:** Hans Gustav Hørsted Thyregod, Troels Højsgaard Jørgensen, Nikolaj Ihlemann, Daniel Andreas Steinbrüchel, Henrik Nissen, Bo Juel Kjeldsen, Petur Petursson, Ole De Backer, Peter Skov Olsen, Lars Søndergaard

**Affiliations:** Department of Cardiothoracic Surgery, The Heart Centre, Rigshospitalet, Copenhagen University Hospital, Blegdamsvej 9, 2100 Copenhagen, Denmark; Department of Cardiology, The Heart Centre, Rigshospitalet, Copenhagen University Hospital, Blegdamsvej 9, 2100 Copenhagen, Denmark; Department of Cardiology, Bispebjerg University Hospital, Bispebjerg Bakke 23, 2400 Copenhagen, Denmark; Department of Cardiothoracic Surgery, The Heart Centre, Rigshospitalet, Copenhagen University Hospital, Blegdamsvej 9, 2100 Copenhagen, Denmark; Department of Cardiology, Odense University Hospital, J. B. Winsløws Vej 4, 5000 Odense, Denmark; Department of Cardiothoracic and Vascular Surgery, Odense University Hospital, J. B. Winsløws Vej 4, 5000 Odense, Denmark; Department of Cardiology, Sahlgrenska University Hospital, Blå Stråket 5, 413 45 Gothenburg, Sweden; Department of Cardiology, The Heart Centre, Rigshospitalet, Copenhagen University Hospital, Blegdamsvej 9, 2100 Copenhagen, Denmark; Department of Cardiothoracic Surgery, The Heart Centre, Rigshospitalet, Copenhagen University Hospital, Blegdamsvej 9, 2100 Copenhagen, Denmark; Department of Cardiology, The Heart Centre, Rigshospitalet, Copenhagen University Hospital, Blegdamsvej 9, 2100 Copenhagen, Denmark

**Keywords:** Aortic valve stenosis, Surgical aortic valve replacement, Transcatheter aortic valve implantation, Transcatheter aortic valve replacement, Bioprosthetic aortic valve durability

## Abstract

**Background and Aims:**

Transcatheter aortic valve implantation (TAVI) has become a viable treatment option for patients with severe aortic valve stenosis across a broad range of surgical risk. The Nordic Aortic Valve Intervention (NOTION) trial was the first to randomize patients at lower surgical risk to TAVI or surgical aortic valve replacement (SAVR). The aim of the present study was to report clinical and bioprosthesis outcomes after 10 years.

**Methods:**

The NOTION trial randomized 280 patients to TAVI with the self-expanding CoreValve (Medtronic Inc.) bioprosthesis (*n* = 145) or SAVR with a bioprosthesis (*n* = 135). The primary composite outcome was the risk of all-cause mortality, stroke, or myocardial infarction. Bioprosthetic valve dysfunction (BVD) was classified as structural valve deterioration (SVD), non-structural valve dysfunction (NSVD), clinical valve thrombosis, or endocarditis according to Valve Academic Research Consortium-3 criteria. Severe SVD was defined as (i) a transprosthetic gradient of 30 mmHg or more and an increase in transprosthetic gradient of 20 mmHg or more or (ii) severe new intraprosthetic regurgitation. Bioprosthetic valve failure (BVF) was defined as the composite rate of death from a valve-related cause or an unexplained death following the diagnosis of BVD, aortic valve re-intervention, or severe SVD.

**Results:**

Baseline characteristics were similar between TAVI and SAVR: age 79.2 ± 4.9 years and 79.0 ± 4.7 years (*P* = .7), male 52.6% and 53.8% (*P* = .8), and Society of Thoracic Surgeons score < 4% of 83.4% and 80.0% (*P* = .5), respectively. After 10 years, the risk of the composite outcome all-cause mortality, stroke, or myocardial infarction was 65.5% after TAVI and 65.5% after SAVR [hazard ratio (HR) 1.0; 95% confidence interval (CI) 0.7–1.3; *P* = .9], with no difference for each individual outcome. Severe SVD had occurred in 1.5% and 10.0% (HR 0.2; 95% CI 0.04–0.7; *P* = .02) after TAVI and SAVR, respectively. The cumulative incidence for severe NSVD was 20.5% and 43.0% (*P* < .001) and for endocarditis 7.2% and 7.4% (*P* = 1.0) after TAVI and SAVR, respectively. No patients had clinical valve thrombosis. Bioprosthetic valve failure occurred in 9.7% of TAVI and 13.8% of SAVR patients (HR 0.7; 95% CI 0.4–1.5; *P* = .4).

**Conclusions:**

In patients with severe AS and lower surgical risk randomized to TAVI or SAVR, the risk of major clinical outcomes was not different 10 years after treatment. The risk of severe bioprosthesis SVD was lower after TAVR compared with SAVR, while the risk of BVF was similar.

## Structured Graphical Abstract

**Structured Graphical Abstract:**
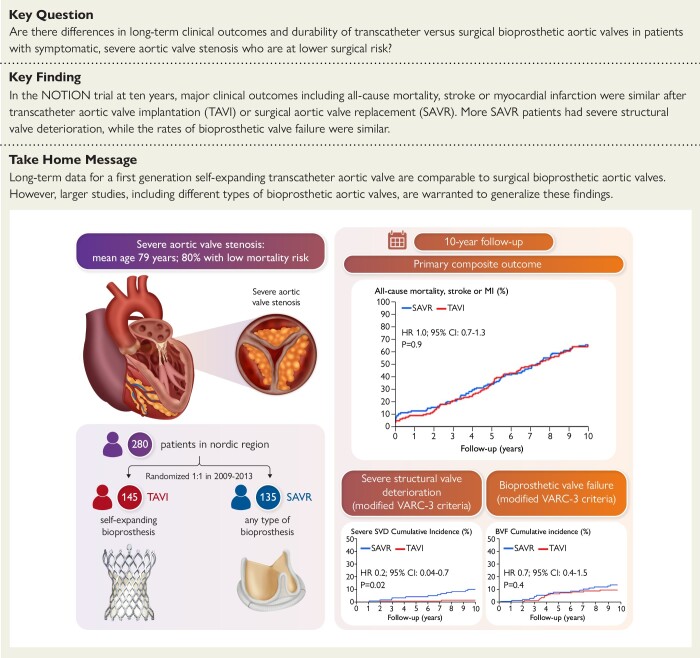
Clinical and bioprosthesis 10-year outcomes after transcatheter or surgical aortic valve implantation. TAVI, transcatheter aortic valve implantation; SAVR, surgical aortic valve replacement. Hazard ratio (HR); 95% confidence interval (CI); the *P*-value was based on Cox regression.

**See the editorial comment for this article ‘Transcatheter vs. surgical treatment of aortic stenosis: long-awaited long-term data, yet a long road to go’, by S. Bleiziffer, https://doi.org10.1093/eurheartj/ehad873.**

## Introduction

Transcatheter aortic valve implantation (TAVI) has revolutionized the treatment of patients with acquired severe aortic valve stenosis (AS). Randomized clinical trials have documented the benefits of TAVI compared with medical therapy in patients who are ineligible for surgery, as well as to surgery in patients who are at high or intermediate surgical risk up to 5 years.^1,2,3,4,5,6^ In recent European guidelines, TAVI is recommended instead of surgery for high-risk and suitable moderate-risk patients and in patients older than 75 years.^7^ Surgery is still recommended for younger and low-risk patients, mainly because the durability of transcatheter heart valves (THV) is unknown. The general recommendation for the use of surgical bioprosthetic aortic valves as opposed to mechanical valves is age older than 65 years, but as TAVI offers a less invasive treatment, an increasing number of younger patients are now treated with THV. In the United States, about half of the patients younger than 65 years treated for isolated AS undergoes TAVI.^8^ Due to the longer life expectancy of these patients, evidence on long-term durability of THV vs. surgical bioprostheses is needed.

The Nordic Aortic Valve Intervention Trial (NOTION) enrolled patients with severe AS from 2009 to 2013 and was the first to randomize primarily patients at lower surgical risk to TAVI or surgical aortic valve replacement (SAVR).^9^ The trial has documented no significant difference in mortality, stroke, or myocardial infarction (MI) up to 8 years after intervention.^10^ Furthermore, no significant difference has been found for structural deterioration of or re-intervention on the bioprostheses used. Two other larger trials including low-risk patients, the PARTNER 3 and Evolut Low Risk trial, have documented outcomes after 5 and 4 years, with no significant difference between TAVI and SAVR for all-cause mortality or disabling stroke.^11,12^ The aim of this secondary analysis was to document clinical and prosthesis outcomes after 10 years in the NOTION trial.

## Methods

The details of the trial design have previously been published.^13^ In short, the trial was investigator-initiated, multicentre, non-blinded, and randomized patients to TAVI or SAVR. Follow-up was yearly and life-long. All patients provided informed written consent. The regional ethical review board approved the protocol at each site, and the trial was conducted according to the principles of the Declaration of Helsinki. All data were collected and stored by the investigators and were externally monitored. Only outcomes within the first post-procedural year were adjudicated by an independent clinical events committee. Outcomes thereafter were adjudicated by the investigators at each centre. Patients suspected of a stroke were evaluated by a neurologist including clinical examination and potentially brain imaging studies. The trial was registered with ClinicalTrials.gov, NCT01057173.

### Patients

All patients aged 70 years or older with symptomatic severe AS were considered for inclusion. No specific surgical risk profile was required if patients were anatomically suitable for both procedures as determined by an echocardiogram and in some cases a computed tomography scan. Major exclusion criteria were the need for acute treatment, other significant cardiovascular diseases, and/or other major organ failures (see Thyregod *et al.*^13^ for more details).

Patients who were randomized to TAVI received a self-expanding first- or second-generation CoreValve bioprosthesis (Medtronic Inc., Minneapolis, MN, USA) using a transfemoral access in almost all cases. Surgical patients had a full sternotomy and standard implantation of a porcine or bovine stented bioprosthesis without the use of annular enlargement techniques. The specific type of bioprosthesis was left at the discretion of the surgeon.

### Outcome definitions

The primary outcome was a composite of all-cause death, stroke, or MI after 1 year. Here, we report the composite outcome and all its components after 10 years. Other relevant clinical outcomes reported include transient ischaemic attack, new-onset atrial fibrillation, permanent pacemaker implantation, and endocarditis. Echocardiographic outcomes were the effective orifice area (EOA) of the bioprosthesis and the mean transprosthetic gradient, the degree of central regurgitation, and paravalvular leakage (PVL). Outcomes were defined according to the Valve Academic Research Consortium (VARC)-2 criteria.^14^

Bioprosthesis durability was classified according to the VARC-3 criteria.^15^ The criteria distinguish between bioprosthetic valve failure (BVF) and bioprosthetic valve dysfunction (BVD). Bioprosthetic valve failure was defined as one of the following three: (i) valve-related death (death caused by BVD or sudden unexplained death following the diagnosis of BVD), (ii) severe haemodynamic structural valve deterioration (SVD), or (iii) prosthesis re-intervention following diagnosis of BVD. Bioprosthetic valve dysfunction was categorized into four groups: (i) SVD (moderate SVD: mean transprosthetic gradient ≥ 20 mmHg and increase ≥ 10 mmHg from 3 months or new ≥ moderate intraprosthetic regurgitation; severe SVD: mean transprosthetic gradient ≥ 30 mmHg and increase ≥ 20 mmHg from 3 months echo or new severe intraprosthetic regurgitation), (ii) non-structural valve deterioration (NSVD) defined as moderate/severe PVL or prosthesis–patient mismatch (PPM) at 3 months [moderate PPM: if body mass index (BMI) < 30 kg/m^2^ then indexed 0.65 cm^2^/m^2^ < indexed EOA ≤ 0.85 cm^2^/m^2^ and if BMI ≥ 30 kg/m^2^ then 0.55 cm^2^/m^2^ < indexed EOA ≤ 0.7 cm^2^/m^2^; severe PPM: if BMI < 30 kg/m^2^ then indexed EOA ≤ 0.65 cm^2^/m^2^ and if BMI ≥ 30 kg/m^2^ then indexed EOA ≤ 0.55 cm^2^/m^2^], (iii) clinical bioprosthetic valve thrombosis, or (iv) endocarditis according to the modified Duke criteria. Prosthesis–patient mismatch was evaluated at the echocardiography performed 3 months post-procedure, whereas SVD was defined as an increase in transprosthetic gradient or intraprosthetic regurgitation over time. The original complete VARC-3 definition of SVD includes a concomitant decrease in EOA and/or a decrease in Doppler velocity index. We used the modified ‘haemodynamic’ definition, as we did not systematically calculate velocity indices.

### Statistics

Clinical outcomes of all-cause mortality, stroke, or MI were reported for the intention-to-treat population. Echocardiographic data and other data on bioprosthesis durability were reported for the as-implanted population. A time-to-event analysis using Kaplan–Meier estimates was used for survival analyses of all-cause mortality. The log-rank test was used for outcome comparisons between treatment groups. When death was a competing risk, the cumulative incidence was analysed using the Aalen–Johansen method, and groups were compared using Gray’s test provided in tables. For echocardiographic outcomes, patients were censored after date of re-intervention if performed. The association between exposure and mortality rates was analysed with Cox regression and reported as hazard ratio (HR) with 95% confidence interval (CI), and the *P*-value in figures was based on cause-specific HR. The association between exposure (TAVI vs. SAVR) and SVD, BVF, and stroke rates was analysed with Cox regression with death (and aortic valve re-intervention in the case of SVD and BVF) considered as competing risk and reported as HR with 95% CI and *P*-value in figures. Furthermore, a multivariate Cox regression including exposure, left ventricular ejection fraction (LVEF) at 3 months post-procedure (≥50% vs. <50%), gender (male vs. female), age (≥ 80 vs. <80 years), presence/absence of pacemaker at 1 month, and/or PVL at 3 months post-procedure analysed the association with all-cause mortality. The association of exposure and atrial fibrillation as a time-dependent variable with the risk of stroke (death considered competing risk) was analysed with Cox regression. All categorical variables were presented as counts and percentages and compared with the χ^2^ or Fisher’s exact test. Continuous variables were presented as mean with standard deviation and compared using Student’s *t*-test or median with interquartile range and compared using the Wilcoxon signed-rank test. The null hypothesis was rejected for *P*-values < .05. All statistical analyses were performed with SAS Enterprise Guide 8.3 (SAS Institute, Cary, NC, USA).

## Results

For the intention-to-treat population, 280 patients were enrolled (145 TAVI and 135 SAVR). There was no significant difference between any baseline characteristic in the two treatment groups for both populations (see Supplementary data online, *Tables S1* and *S2*). The mean age was 79.1 ± 4.8 years, 47% were female, and mean Society of Thoracic Surgeons Predicted Risk of Mortality (STS-PROM) score was 3.0 ± 1.7%, indicating a low-risk cohort. Four patients died before receiving a procedure, 3 TAVI patients crossed over to SAVR after attempted TAVI, and 3 SAVR patients never received a bioprosthesis, leaving 274 patients (139 TAVI and 135 SAVR) for the as-implanted population (see Supplementary data online, *Figure S1*). After 10 years, 98.9% of patients could be followed up (2 TAVI and 1 SAVR patients were lost) and of these 101 (36.1%) patients were alive. Echocardiographic data were available for 82 (81.2%) patients reaching 10 years. Missing 10-year echo data for 12 of 52 (23.1%) TAVI and 7 of 49 (14.2%) SAVR patients. For procedural details, see Supplementary data online, *Tables S1*, *S2*, and *S4*.

### Clinical outcomes

**Figure 1:**
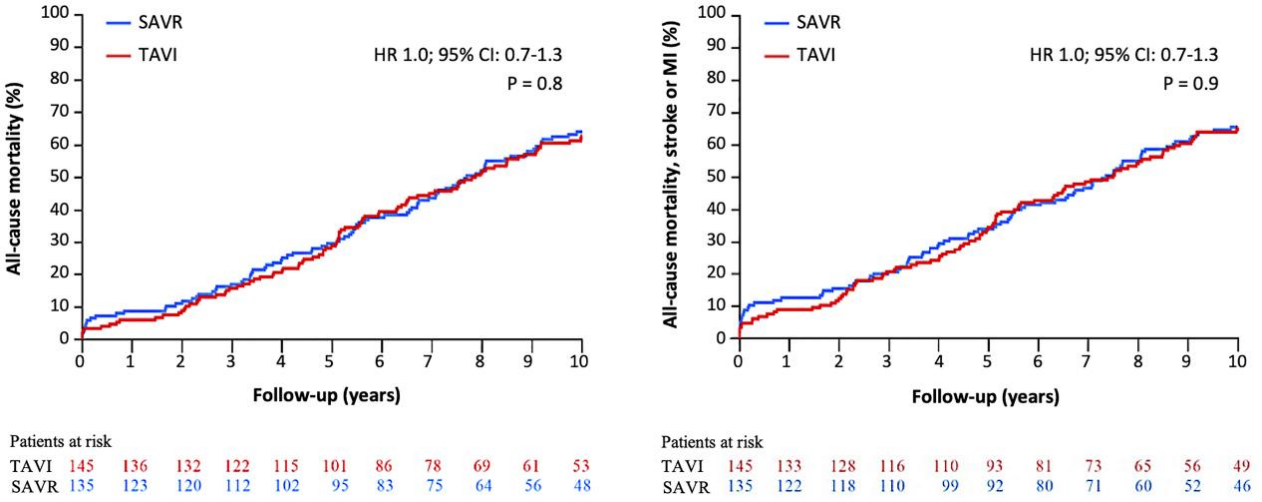
NOTION—clinical outcomes up to 10 years of follow-up: all-cause mortality and all-cause mortality, stroke, or myocardial infarction (MI). TAVI, transcatheter aortic valve implantation; SAVR, surgical aortic valve replacement; MI, myocardial infarction. Hazard ratio (HR); 95% confidence interval (CI); *P*-value was based on Cox regression

After 10 years in the intention-to-treat population, there was no difference for all-cause mortality between the two treatment groups (TAVI 62.7% and SAVR 64.0%, HR 1.0; 95% CI 0.7–1.3, *P* = .8) (*Figure 1*). An analysis for all-cause mortality in three age groups can be found the Supplementary data online, *Figure S2*. For the initial primary composite outcome of all-cause mortality, stroke, or MI, no difference could be found for this composite outcome (TAVI 65.5% and SAVR 65.5%, HR 1.0; 95% CI 0.7–1.3, *P* = .9). Furthermore, for each component in the composite outcome, no difference could be found (*Table 1*). About half of MIs occurred immediately after the procedure and were rarely confirmed by angiographic studies. More SAVR patients had experienced new-onset atrial fibrillation (TAVI 52.0% and SAVR 74.1%, *P* < .01) at any time during follow-up. At 10 years, 13.0% of TAVI and 20.5% of SAVR patients (*P* = .4) had atrial fibrillation on their ECG. For anticoagulation therapy, see Supplementary data online, *Table S3*. More TAVI patients without a pacemaker at baseline had received a new permanent pacemaker (TAVI 44.7% and SAVR 14.0%, *P* < .01) (*Table 1*), with the majority of implants occurring during the first year after the procedure.^9^ Similar results were found in the as-implanted population. In the multivariate Cox regression analysis, only age older than 80 years was significantly associated with increased all-cause mortality after 10 years (see Supplementary data online, *Table S5*). Permanent pacemaker implantation during the first 30 days after the index procedure or moderate/severe PVL was not associated with all-cause mortality. Type of procedure or presence of atrial fibrillation was not associated with the risk of stroke (see Supplementary data online, *Table S6*). The distribution of New York Heart Association (NYHA) functional class for those alive at 10 years was similar between groups (NYHA classes I and II, TAVI 83.7% and SAVR 80.6%, *P* = .93) (see Supplementary data online, *Figure S3*).

**Table 1:** Adverse outcomes

	TAVI	SAVR	*P*-value
	(*n* = 145)	(*n* = 135)	
All-cause mortality	62.7	64.0	.8
Cardiovascular death	49.5	51.2	.7
Stroke^a^	9.7	16.4	.1
Stroke with sequelae	6.9	10.4	.3
Transient ischaemic attack	9.7	6.7	.3
Myocardial Infarction	11.0	8.2	.4
New-onset atrial fibrillation	52.0	74.1	<.01
New permanent pacemaker	44.7	14.0	<.01

All analyses are cumulative incidence compared with Gray’s test, except the absolute risk of all-cause mortality compared with log-rank test. Risk estimates are %.

TAVI, transcatheter aortic valve implantation; SAVR, surgical aortic valve replacement.

^a^Include both disabling and non-disabling strokes.

### Echocardiographic outcomes

**Figure 2:**
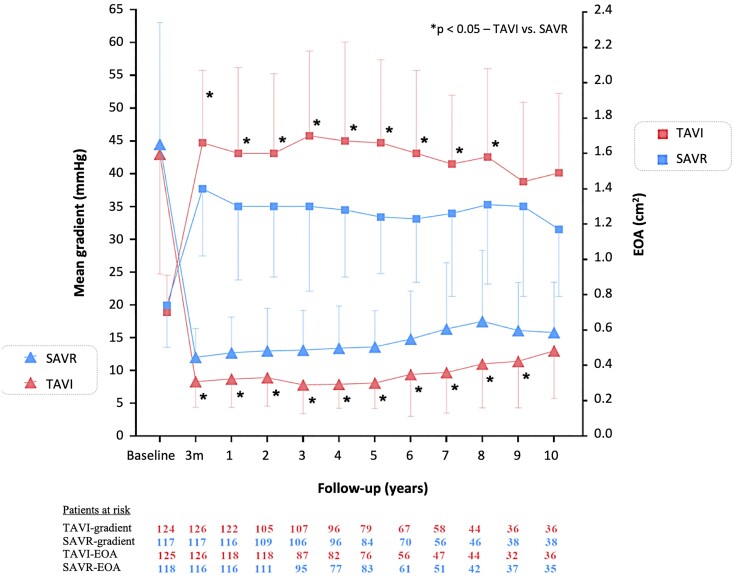
NOTION—aortic valve haemodynamics up to 10 years of follow-up: transprosthetic gradient and effective orifice area (EOA). EOA, effective orifice area; TAVI, transcatheter aortic valve implantation; SAVR, surgical aortic valve replacement. *P < .05 for inter-group comparison

The initial improvement in EOA and corresponding decrease in mean transprosthetic gradient seen after both procedures remained significant within groups, but over time, EOA decreased and the gradient increased for both groups (*Figure 2*). At all time-points, the increase in area and decrease in gradient was more pronounced for TAVI compared with SAVR. Transcatheter aortic valve implantation patients had more moderate or severe PVL or bioprosthesis regurgitation after 10 years (risk of moderate/severe PVL at any time during follow-up, TAVI 25.4% and SAVR 2.5%, *P* < .01). For TAVI patients, there was no association between moderate or severe PVL at 3 months after the procedure and all-cause mortality after 10 years (rate of all-cause mortality for moderate/severe PVL 62.0% and no/mild PVL 55.0%, *P* = .84) (see Supplementary data online, *Table S5*). No difference in LVEF after 10 years could be found between the two groups (TAVI 50.7% and SAVR 52.8%, *P* = .11).

### Bioprosthesis durability

**Figure 3:**
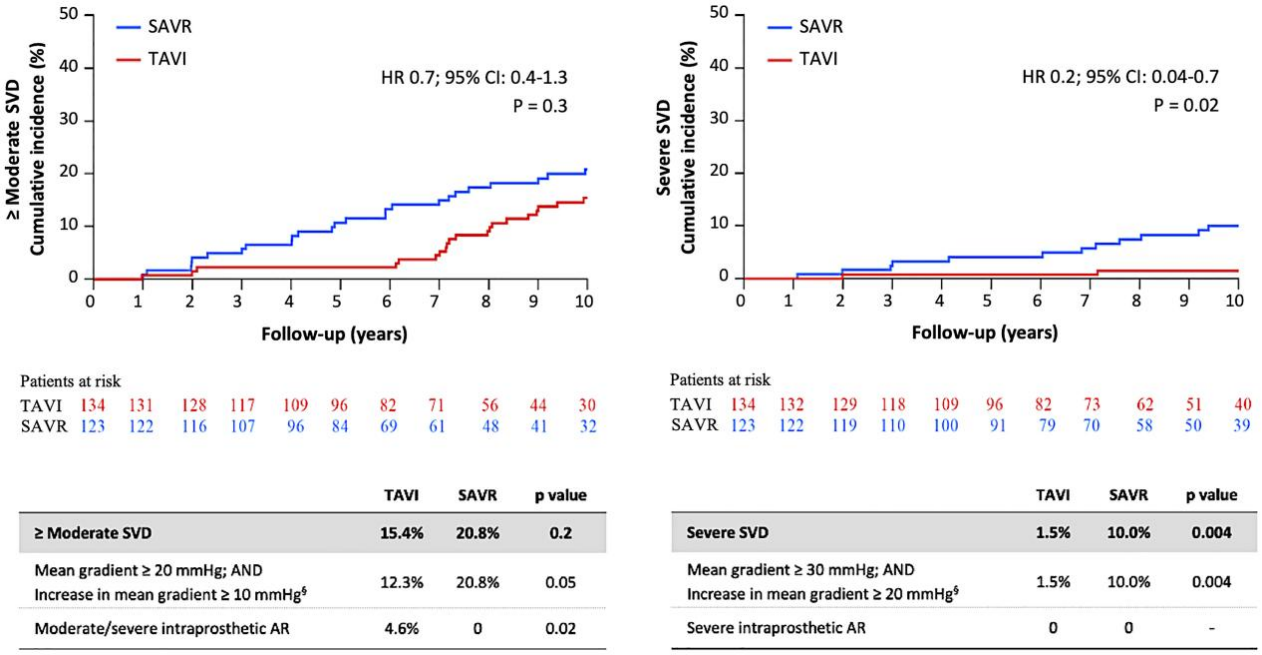
Structural valve deterioration (SVD)—haemodynamic VARC-3 definition: ≥moderate SVD and severe SVD. SVD, structural valve deterioration; AR, aortic valve regurgitation; TAVI, transcatheter aortic valve implantation; SAVR, surgical aortic valve replacement; VARC-3, Valve Academic Research Consortium third definition. Table and curve are cumulative incidences after 10 years of follow-up compared with Gray’s test. Hazard ratio (HR); 95% confidence interval (CI); *P*-value was based on Cox regression

**Figure 4:**
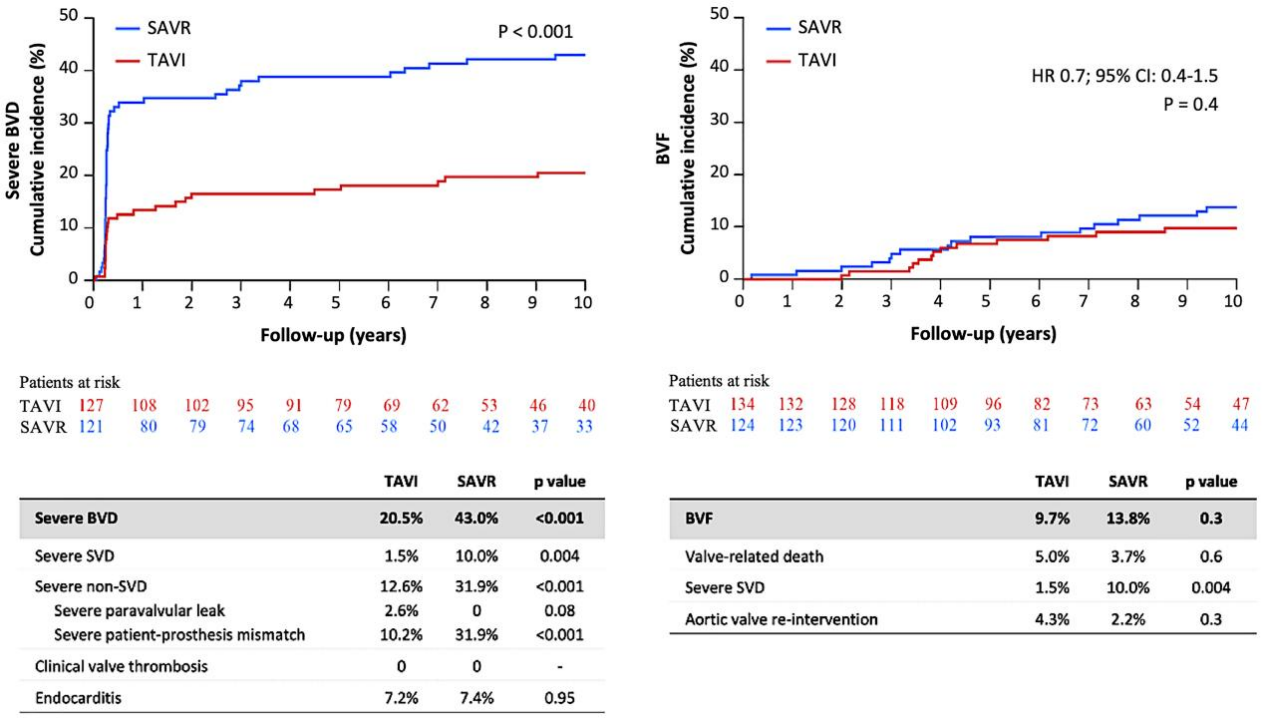
Bioprosthetic valve dysfunction (BVD) and failure (BVF)—haemodynamic VARC-3 definition: severe BVD and BVF. BVD, bioprosthetic valve dysfunction; BVF, bioprosthetic valve failure; TAVI, transcatheter aortic valve implantation; SAVR, surgical aortic valve replacement. VARC-3, Valve Academic Research Consortium third definition. Table and curve are cumulative incidences after 10 years of follow-up compared with Gray’s test. Hazard ratio (HR); 95% confidence interval (CI); *P*-value was based on Cox regression

In the as-implanted population after 10 years, the risk of moderate or severe SVD was similar after TAVI compared with SAVR (TAVI 15.4% and SAVR 20.8%, HR 0.7; 95% CI 0.4–1.3, *P* = .3) (*Figure 3*), while the risk of severe SVD was lower for TAVI (TAVI 1.5% and SAVR 10.0%, HR 0.2; 95% CI 0.04–0.7, *P* = .02). The proportion of patients with mild or more PVL at 10 years was larger for TAVI than SAVR (TAVI 18.0% and SAVR 5.2%, *P* < .05), while the proportion with mild or moderate intraprosthetic regurgitation was similar between groups (TAVI 5.8% and SAVR 2.2%, *P* = .5) (see Supplementary data online, *Figures S4–S6*). Severe BVD occurred less frequent in the TAVI group compared with SAVR (TAVI 20.5% and SAVR 43.0%, *P* < .01) (*Figure 4*). This was mainly driven by a high risk of severe NSVD, e.g. severe PPM, for SAVR patients (TAVI 10.2% and SAVR 31.9%, *P* < .01). No patient had clinical valve thrombosis, and the rate of infective endocarditis was low and similar for both groups (TAVI 7.2% and SAVR 7.4, *P* = 1.0). The risk of BVD over time within each group was similar. Overall, there was no difference in BVF between groups (TAVI 9.7% and SAVR 13.8%, HR, 0.7; 95% CI: 0.4–1.5, *P* = .4), and in particular, the rate of prosthesis re-intervention was low and similar for the two types of bioprostheses (TAVI 4.3% and SAVR 2.2%, *P* = .3). Reasons for re-intervention were restenosis (five TAVI and two SAVR) and central regurgitation (1 TAVI and 1 SAVR), and TAVI was done for all re-interventions. For cumulative incidences of SVD, BVD, and BVF defined according to VARC-3 but excluding Doppler velocity index, see Supplementary data online, *Figures S7* and *S8*.

## Discussion

The NOTION trial completed enrolment in 2013 and included patients at lower surgical risk. This allows for the first comparison of clinical outcomes and durability after TAVI or SAVR with 10-year follow-up. We found no significant difference for all-cause mortality, stroke, or MI. As seen in our previous trial reports, new-onset atrial fibrillation occurred more often after SAVR in the immediate post-operative period. More TAVI patients had conduction abnormalities immediately after the procedure requiring a permanent pacemaker. The rates of bioprosthesis endocarditis and re-intervention were very low and similar between the two groups. The bioprosthetic EOA was larger after TAVI, but more patients had PVL compared with SAVR patients. The risk of SVD and BVF was not different for the two types of bioprostheses (Structured Graphical Abstract).

### Clinical outcomes

Most trials and observational studies comparing TAVI with SAVR beyond 5 years have included old and moderate- and high-risk patients resulting in high overall mortality rates, caused by both non- and cardiovascular causes, and therefore small study populations at risk. All-cause mortality rates after 5 years were similar between TAVI and SAVR (55.3%–67.8% for TAVI and 55.4%–62.4% for SAVR) in high- and intermediate-risk patients (30%–46% for TAVI and 28.7%–42.1% for SAVR).^2,3,5,6,^ In the NOTION trial, all consecutive patients were considered for enrolment and no specific risk profile was defined. This resulted in the enrolment of old, mean age 79 years, but lower-risk patients, with more than 80% of patients having a STS-PROM score < 4% and limited comorbidities. Frailty was not systematically assessed. A recent observational study in low-risk patients found an all-cause mortality rate of 7.1% and 12.4% 5 and 8 years after isolated SAVR, respectively.^16^ These rates were 28.9% and 52.6% in our trial.^10,17^ After 10 years, more than 60% of patients had died, suggesting a higher risk profile than the one measured by the STS-PROM score, while the type of intervention and bioprosthesis did not seem to affect the outcome. The two other trials in younger low-risk patients currently only have intermediate-term follow-up with all-cause mortality rates of 10.0% for TAVI and 8.2% for SAVR after 5 years in the PARTNER 3 trial^11^ and 9.0% for TAVI and 12.1% for SAVR after 4 years in the Evolut Low Risk trial,^12^ with no significant difference in either trial. Mean age in these larger trials were 73 and 74 years, respectively, and up to 26% of surgical patients underwent concomitant cardiac procedures including coronary artery bypass grafting. Similar rates of disabling stroke (5.8% and 2.9% for TAVI; 6.4% and 3.8% for SAVR) and cardiac re-hospitalizations were reported, but more SAVR patients had new-onset atrial fibrillation and serious bleeding. For TAVI patients in the Evolut Low Risk trial, using a self-expanding THV, the rate of pacemaker implantation was higher compared with SAVR patients. We also found less new-onset atrial fibrillation but more pacemaker implantation after TAVR compared with SAVR but at higher rates.

Procedural factors such as conduction abnormalities and all degrees of PVL and PPM have all been associated with an increased risk of death in both trials and observational studies.^3,4,18,19,20,21,22^ We have not been able to demonstrate any of these associations in the NOTION trial, although our rates of these complications were higher compared with more contemporary trials.^9,23^ The limited sample size could be an explanation for this finding.

### Bioprosthesis haemodynamics and durability

After both interventions, the EOA increased significantly and significantly more after TAVI compared with SAVR. Consequently, the transprosthetic gradient was lower after TAVI. This did not result in differences in LVEF or left ventricular mass regression as reported previously.^24^ These more favourable haemodynamic parameters after TAVI have been demonstrated in all trials. On the other hand, significantly more TAVI patients had PVL. The rate of mild or more PVL after TAVI was higher in the NOTION trial (53% after 5 years)^17^ compared with more recent trials using newer delivery systems and THV designs (15.3% and 20.8% after 4 and 5 years, respectively).^11,12^ More than 98% of TAVI patients had a THV sized 26–31 mm, while 98% of SAVR patients received a size 19–25 mm stented bioprosthesis from various manufacturers. Consequently, more SAVR patients had PPM and high transprosthetic gradients. This explains the higher rates of NSVD, as PPM (e.g. the prosthetic opening area indexed to body size 3 months after the procedure) is part of the NSVD definition. We did not observe any other NSVD such as cusp entrapment by pannus, dilatation of the aortic root, prosthesis erosion, or embolization. The rate of severe SVD was higher after SAVR (e.g. a significant change in prosthetic cusp structure leading to an increase in transprosthetic gradient). No patients had severe intraprosthetic regurgitation. The ‘haemodynamic’ VARC-3 definition of SVD used in the current report included only the transprosthetic gradient and has been shown to be more predictive of adverse clinical outcomes than the complete VARC-3 definition.^25^ Both the calculated EOA and Doppler velocity index, included in the complete definition, are more affected by observer variability and error.^26^ We found no difference in severe SVD if EOA was included in the definition. A too small prosthesis causing a high transprosthetic gradient and persistent left ventricular hypertrophy has been associated with an accelerated risk of SVD, heart failure, and decreased survival.^20,21,22^ Other clinical predictors of SVD have been reported to be a younger age, smoking, higher BMI, dyslipidaemia, diabetes mellitus, and renal insufficiency. Furthermore, the specific type of bioprosthesis used for TAVR, e.g. self-expanding or balloon-expanding, and SAVR, e.g. pericardial or porcine and externally or internally mounted cusps, will also influence the rate of SVD and should be considered when comparing trial results.^27^ We used five different surgical bioprostheses (both bovine pericardial and porcine and with internally or externally mounted cusps on the stent) making comparisons with other study cohorts difficult. In the PARTNER 2A trial, the rate of SVD after 5 years was higher after TAVR using the SAPIEN-XT balloon-expanding THV compared with SAVR using a pericardial bioprosthesis in 80% of patients (1.61 ± 0.24% vs. 0.63 ± 0.16%, *P* ≤ .01), while the SVD rate for the SAPIEN 3 THV was not different.^11,28^ For the self-expanding THV, the cumulative incidence of SVD using the ‘haemodynamic’ VARC-3 criteria was lower after TAVR compared with SAVR after 5 years (2.20% vs. 4.38%; HR, 0.46; 95% CI, 0.27–0.78; *P* = .004).^25^ The rates of BVF, in particular bioprosthesis re-intervention, and endocarditis were similar between groups. Causes of re-intervention were primarily stenosis of the prosthesis, and re-intervention with TAVI was used for all cases. The rates of endocarditis and causative bacteria were similar to findings from other series.^29^ In the PARTNER 3 trial using a balloon-expanding THV, clinically significant bioprosthesis thrombosis occurred more often in TAVI patients.^11^ We did not find any clinically significant bioprosthesis thrombosis or late coronary obstruction in our trial. There was no systematic screening for subclinical leaflet thrombosis or thickening with high-resolution cardiac computed tomography scans.^30^ As described above, we have not experienced any signs of earlier failure nor improved durability for THV compared with surgical bioprostheses after 10 years. This emphasizes the concept of lifetime management when considering treatment of young and/or lower-risk AS patients with few comorbidities, higher risk of a bicuspid aortic valve, and longer life expectancy.^31^ Conduction abnormalities and even mild PVL could negatively impact survival making the evaluation of native aortic valve anatomy and size, the choice of THV, and its positioning even more important. Bioprosthesis re-intervention will probably be required, but the optimal type of re-intervention is not known at this time. Furthermore, coronary access is compromised in some patients after TAVI and even more after redo TAVI,^32,33^ and for patients younger than 65 years of age, observational studies have demonstrated a survival benefit of surgical mechanical prostheses compared with biological prostheses.^34^ Despite of the lack of evidence for the use of TAVI in younger and/or lower-risk patients, its use has substantially increased in both the USA and Europe.^8,35^

### Trial limitations

The NOTION trial was designed with a primary outcome after the first year; consequently, all other analyses are considered exploratory. More SAVR patients had withdrawn from the trial, and this could have introduced attrition bias. All outcome assessments were done unblinded. Both echocardiograms and strokes were site reported. All patients with concomitant significant cardiac diseases, primarily coronary artery disease, and bicuspid aortic valves were excluded, and results cannot be extrapolated to these patients. We only used transthoracic echocardiography for trial screening and THV sizing. Current guidelines recommend concurrent computed tomography scans to improve THV sizing and positioning.^7,36^ We only used one type of an early generation delivery system and self-expanding THV. Currently available multiple different types of THVs have different designs and delivery systems with proven lower rates of PVL, conduction abnormalities, and vascular complications.^37,38^ For SAVR, no annular enlargement techniques were used to increase bioprosthesis sizes. Surgical bioprostheses with externally mounted leaflets on the stent and now known decreased durability were used,^39,40^ and only 10% of SAVR patients received a pericardial bioprosthesis which has demonstrated improved durability compared with porcine bioprostheses.^41^

## Conclusions

The NOTION trial randomized lower-risk patients with severe AS to TAVI vs. SAVR. After 10 years, no significant differences were found between the two groups regarding all-cause mortality, stroke, or MI. More TAVR patients had permanent pacemaker implantation. Surgical aortic valve replacement patients had new-onset atrial fibrillation more often. Transcatheter aortic valve implantation patients had larger EOA and lower transprosthetic gradients but more PVL. The rate of severe SVD was higher after SAVR but was not significantly different for BVF. Rates of re-intervention were very low and not different between groups. More long-term follow-up data from trials are required to recommend one type of intervention over the other in lower-risk AS patients.

## Supplementary Material

ehae043_Supplementary_Data

## Data Availability

Due to the nature of this research, participants of this study did not agree for their data to be shared publicly, therefore supporting data are not available.

## Pre-registered Clinical Trial Number

The trial was registered with ClinicalTrials.gov, NCT01057173.
