# Supplementary material for: Transcatheter or surgical aortic valve implantation: 10-year outcomes of the NOTION trial
Source: Eur Heart J. 2024 Feb 7;45(13):1116–24. doi: 10.1093/eurheartj/ehae043 (PMC10984572; doi:10.1093/eurheartj/ehae043)
Supplement: ehae043_Supplementary_Data [file ehae043_supplementary_data.docx]

**Supplementary material**

**Supplementary Table 1 –** Baseline characteristics of the Intention-to-treat population

|  | **TAVI**  **(n = 145)** | **SAVR**  **(n = 135)** |
| --- | --- | --- |
| **Baseline** |  |  |
| Age, Years | 79.2 (4.9) | 79.0 (4.7) |
| Male | 78 (53.8%) | 71 (52.6%) |
| STS-PROM Score | 2.9 (1.6) | 3.1 (1.7) |
| Diabetes Mellitus | 26 (17.9%) | 28 (20.7%) |
| Hypertension | 103 (71.0%) | 103 (76.3%) |
| Peripheral Vascular Disease | 6 (4.1%) | 9 (6.7%) |
| Cerebrovascular incidence | 24 (16.6%) | 22 (16.3%) |
| Chronic lung Disease | 17 (11.7%) | 16 (11.9%) |
| NYHA |  |  |
| I | 7/144 (4.9%) | 3/134 (2.2%) |
| II | 67/144 (46.5%) | 70/134 (52.2%) |
| III | 67/144 (46.5%) | 57/134 (42.5%) |
| IV | 3/144 (2.1%) | 4/134 (3.0%) |
| Cardiac Risk factors |  |  |
| Prior PCI | 11 (7.6%) | 12 (8.9%) |
| Pre-existing pacemaker | 5 (3.4%) | 6 (4.4%) |
| Prior Myocardial infarction | 8 (5.5%) | 6 (4.4%) |
| Known atrial fibrillation/flutter | 40/144 (27.8%) | 34/133 (25.6%) |
| **Procedure** |  |  |
| Procedure Time* | 90.3 (38.6) | 177.2 (39.8) |
| Procedural Success | 139/142 (97.9%) | NA |
| Local Anaesthesia | 26/142 (18.3%) | NA |
| Implantation of >1 valve | 4/142 (2.8%) | NA |
| Conversion to other procedure | 3/142 (2.1%) | 2/134 (1.5%) |
| Transfemoral access | 137/142 (96.5%) | NA |
| Subclavian access | 5/142 (3.5%) | NA |
| Valve sizes |  |  |
| 19mm |  | 11/132 (8.3%) |
| 21mm |  | 42/132 (31.8%) |
| 23mm | 2/142 (1.4%) | 45/132 (34.1%) |
| 25mm |  | 32/132 (24.2%) |
| 26mm | 57/142 (40.1%) |  |
| 27mm |  | 2/132 (1.5%) |
| 29mm | 69/142 (48.6%) |  |
| 31mm | 14/142 (9.9%) |  |

Mean (standard deviation) or counts (percentage)

NYHA: New York Heart Association functional class; PCI: Percutaneous coronary intervention; SAVR: Surgical aortic valve replacement; STS-PROM: Society of Thoracic Surgeons Predicted Risk of Mortality; TAVI: Transcatheter aortic valve implantation

* p < 0.05

**Supplementary Table 2 –** Baseline characteristics of as-implanted population

|  | **TAVI**  **(n = 135)** | **SAVR**  **(n = 139)** |
| --- | --- | --- |
| **Baseline** |  |  |
| Age, Years | 79.4 (4.9) | 78.8 (4.6) |
| Male | 73 (52.5%) | 72 (53.3%) |
| STS-PROM Score | 2.9 (1.7) | 2.9 (1.6) |
| Diabetes Mellitus | 24 (17.3%) | 28 (20.7%) |
| Hypertension | 100 (71.9%) | 105 (77.8%) |
| Peripheral Vascular Disease | 6 (4.3%) | 9 (6.7%) |
| Cerebrovascular incidence | 22 (15.8%) | 22 (16.3%) |
| Chronic lung Disease | 17 (12.2%) | 16 (11.9%) |
| NYHA |  |  |
| I | 7/134 (5.0%) | 3/138 (2.4%) |
| II | 67/134 (48.2%) | 69/138 (51.1%) |
| III | 62/134 (44.6%) | 58/138 (43.0%) |
| IV | 4/134 (3.0%) | 2/138 (1.5%) |
| Cardiac Risk factors |  |  |
| Prior PCI | 11 (7.9%) | 12 (8.9%) |
| Pre-existing pacemaker | 6 (4.3%) | 6 (4.4%) |
| Prior Myocardial infarction | 8 (5.8%) | 6 (4.4%) |
| Known atrial fibrillation/flutter | 92 (66.2%) | 91 (67.4%) |
| **Procedure** |  |  |
| Procedure Time – minutes* | 89.1 (38.1) | 177.4 (38.8) |
| Local Anesthesia | 114 (82.0%) | NA |
| Implantation of >1 valve | 3 (2.2%) | NA |
| Transfemoral access | 134 (96.4%) | NA |
| Subclavian access | 5 (3.6%) | NA |
| Valve sizes |  |  |
| 19mm |  | 11 (8.2%) |
| 21mm |  | 42 (31.1%) |
| 23mm | 2 (1.4%) | 46 (34.1%) |
| 25mm |  | 33 (24.4%) |
| 26mm | 57 (41.0%) |  |
| 27mm |  | 3 (2.2%) |
| 29mm | 68 (48.9%) |  |
| 31mm | 12 (8.6%) |  |

Mean (standard deviation) or counts (percentage)

NYHA: New York Heart Association functional class; PCI: Percutaneous coronary intervention; SAVR: Surgical aortic valve replacement; STS-PROM: Society of Thoracic Surgeons Predicted Risk of Mortality; TAVI: Transcatheter aortic valve implantation

* p < 0.05

**Supplementary Table 3 –** Anti-thrombotic therapy and atrial fibrillation at the time of the specified follow-up

|  | **At 1 month of follow-up** | **1 year of follow-up** | **5 years of follow-up** | **10 years of follow-up** |
| --- | --- | --- | --- | --- |
| **TAVI** | (n = 140) | (n = 136) | (n = 103) | (n = 55) |
| Pre- and post-procedural  Atrial fibrillation | 44.3 | 56.6 | 59.2 | 56.3 |
| Anticoagulant (warfarin, NOAC) | 19.3 | 27.1 | 21.1 | 7.8 |
| Acetylsalicylic acid | 82.1 | 84.2 | 68.9 | 47.1 |
| Other antiplatelet therapy | 94.3 | 12.0 | 10.0 | 13.7 |
| Dual anti-thrombotic therapy | 95.0 | 26.3 | 10.0 | 0 |
| Triple anti-thrombotic therapy | 0.7 | 0 | 1.1 | 0 |
| **SAVR** | (n = 128) | (n =123) | (n = 95) | (n = 49) |
| Pre- and post-procedural  Atrial fibrillation | 75.8 | 78.9 | 79.0 | 83.7 |
| Anticoagulant (warfarin, NOAC) | 39.3 | 31.9 | 31.8 | 11.9 |
| Acetylsalicylic acid | 73.5 | 73.1 | 47.1 | 38.1 |
| Other antiplatelet therapy | 59.1 | 15.1 | 21.2 | 11.9 |
| Dual anti-thrombotic therapy | 68.9 | 23.5 | 12.9 | 0 |
| Triple anti-thrombotic therapy | 3.0 | 0 | 0 | 0 |

Percentages.

NOAC: non-vitamin K anticoagulation; SAVR: surgical aortic valve replacement; TAVI: transcatheter aortic valve implantation

**Supplementary Table 4** – Sizes and type of surgical and transcatheter bioprostheses implanted

| **Surgical bioprostheses** | **19mm**  **(n = 11)** | **21mm**  **(n = 42)** | **23mm**  **(n = 46)** | **25mm**  **(n = 33)** | **27mm**  **(n = 3)** | **All**  **(n = 135)** | 10-year  SVD (VARC3)  (95%CI)  [P-value, vs. CoreValve] |
| --- | --- | --- | --- | --- | --- | --- | --- |
| Mosaic | 2  (18.2) | 12  (28.6) | 8  (17.4) | 14  (42.4) | 1  (33.3) | 37  (27.4) | 17.5  (7.0 – 32-1)  [0.4] |
| Epic | 3  (27.3) | 11  (26.2) | 17  (37.0) | 7  (21.2) | 1  (33.3) | 39  (28.9) | 8.1  (2.0 – 20.1)  [0.4] |
| Trifecta | 6  (54.5) | 11  (26.2) | 11  (23.9) | 3  (9.1) | 1  (33.3) | 32  (23.7) | 7.4  (1.2 – 21.4)  [0.5] |
| Perimount | 0 | 6  (14.3) | 6  (13.0) | 2  (6.1) | 0 | 14  (10.4) | 0  ( - )  [0.2] |
| Sorin mitroflow | 0 | 2  (4.8) | 4  (8.7) | 7  (21.2) | 0 | 13  (9.6) | 30.0  (5.3 – 61.1)  [0.08] |
| **Transcatheter heart valve** | **23mm** | **26mm** | **29mm** | **31mm** |  |  |  |
| CoreValve sizes  (n = 139) | 2  (1.4) | 53  (38.1) | 59  (42.4) | 11  (7.9) |  |  | 13.1  (8.0 – 19.6) |

Counts (percentage).

For structural valve deterioration (SVD with VARC-3 definition) is shown cumulative incidence with 95% confidence interval (95%CI) and compared with Gray’s test.

**Supplementary Table 5** – Multivariate Cox regression of all-cause mortality.

| Covariate | Hazard Ratio | 95% Confidence interval | p-value |
| --- | --- | --- | --- |
| TAVI vs SAVR | 0.9 | 0.6 – 1.3 | 0.5 |
| Male gender | 0.8 | 0.6 – 1.1 | 0.8 |
| Age >80 years at baseline | 2.0 | 1.5 – 2.8 | <0.0001 |
| Pacemaker at 1-month post-TAVI/SAVR | 1.5 | 1.0 – 2.2 | 0.07 |
| LVEF ≥50 at 3 months Echo | 0.9 | 0.6 – 1.3 | 0.5 |
| Moderate/severe PVL at 3 months Echo | 1.0 | 0.5 – 1.9 | 0.9 |

LVEF: left ventricular ejection fraction; PVL paravalvular leakage; SAVR: Surgical aortic valve replacement; TAVI: Transcatheter aortic valve implantation

**Supplementary Table 6** – Multivariate Cox regression of stroke.

| Covariate | Hazard Ratio | 95% Confidence interval | p-value |
| --- | --- | --- | --- |
| TAVI vs SAVR | 0.8 | 0.3 – 1.7 | 0.5 |
| Atrial fibrillation (time-dependent variable) | 2.1 | 0.8 – 5.8 | 0.1 |

SAVR: Surgical aortic valve replacement; TAVI: Transcatheter aortic valve implantation

**Supplementary Figure 1** – Study population


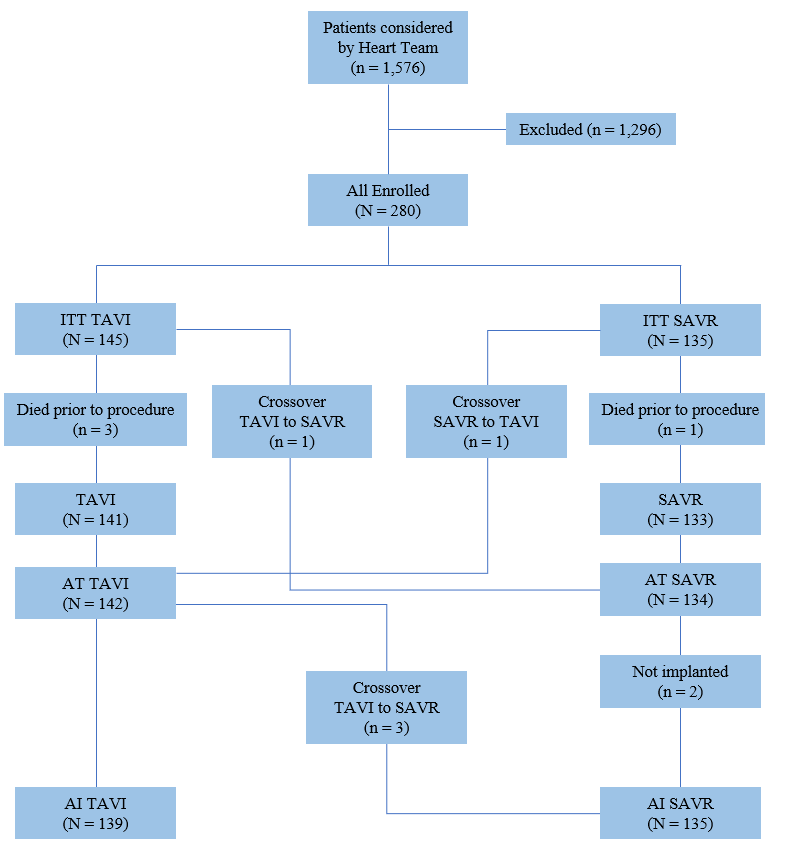


AI: as-implanted population; AT: as-treated population; ITT: intention-to-treat population; SAVR: Surgical aortic valve replacement; TAVI: Transcatheter aortic valve implantation

**Supplementary Figure 2** – All-cause mortality according to age

**
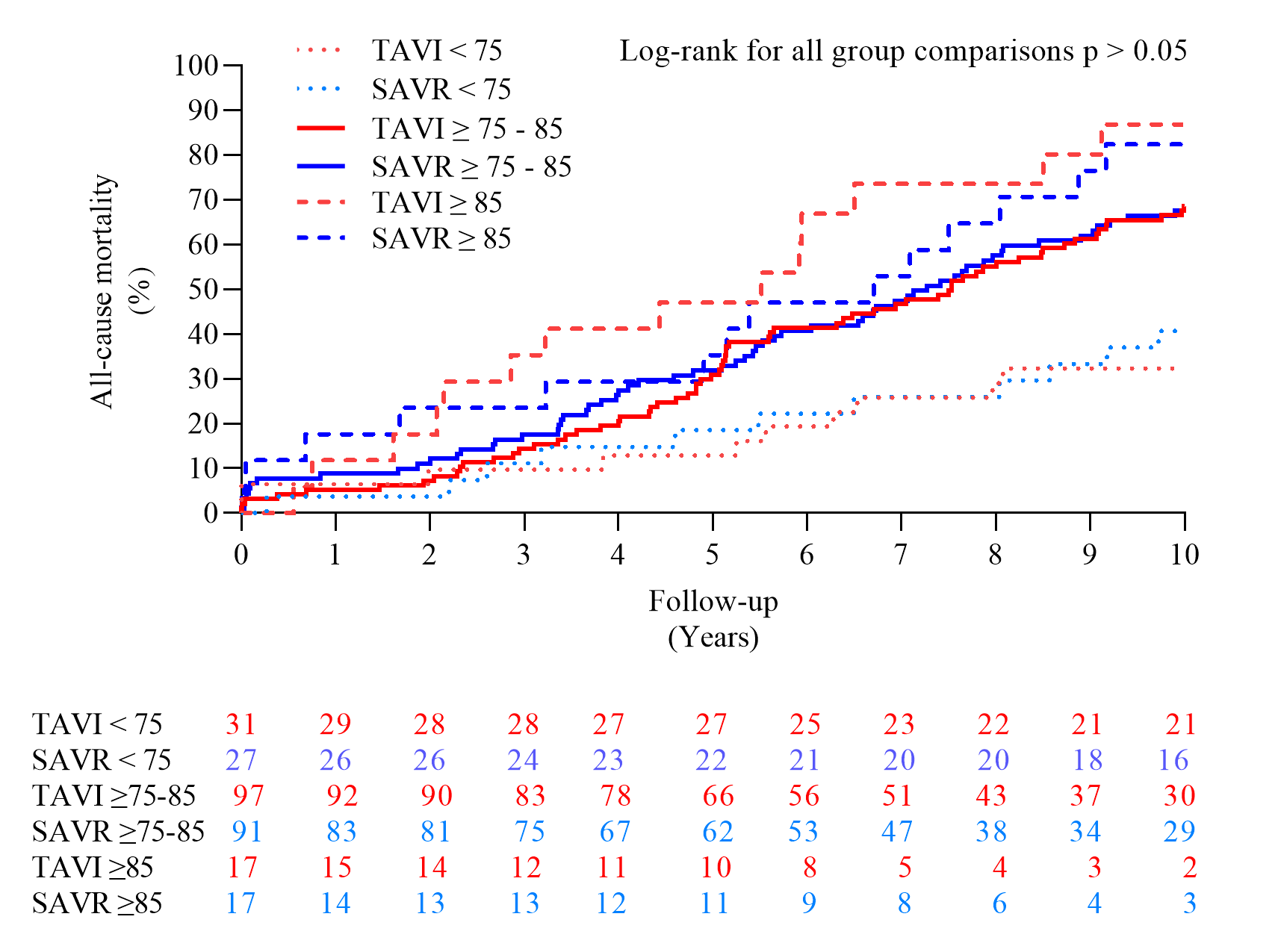
**

A time-to-event analysis using Kaplan-Meier estimates for three age strata (70-74, 75-84, >85 years). The log-rank test was used for comparisons between treatment groups. TAVI, transcatheter aortic valve implantation; SAVR, surgical aortic valve replacement.

**Supplementary Figure 3 –** Distribution of NYHA functional Class


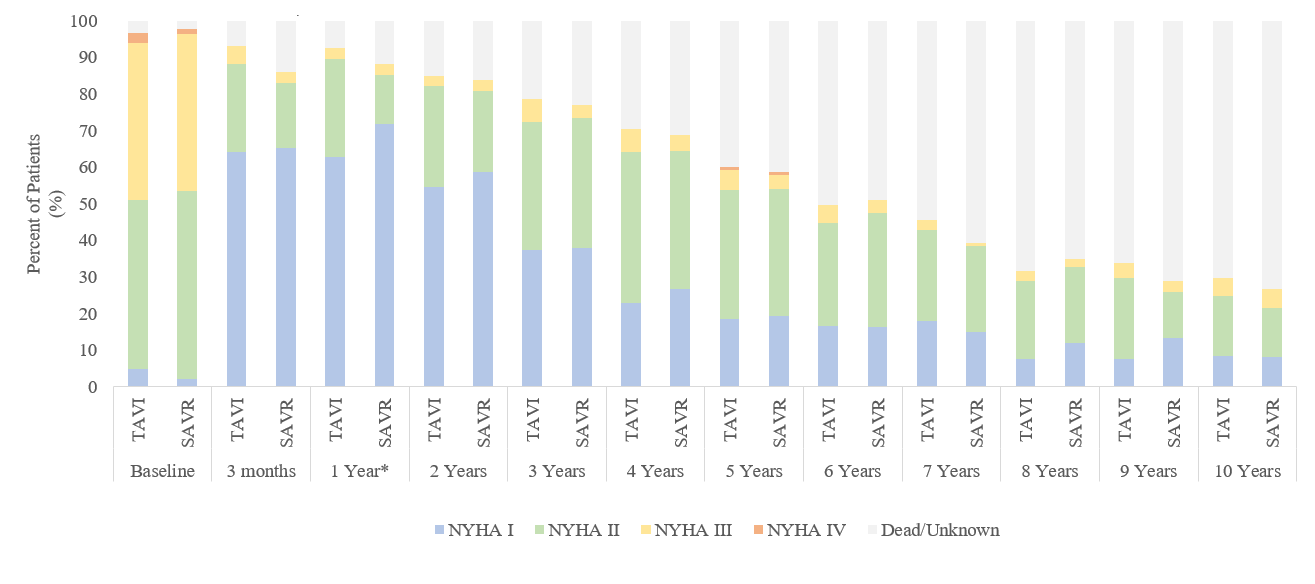


NYHA: New York Heart Association functional class; SAVR: Surgical aortic valve replacement; TAVI: Transcatheter aortic valve implantation

*p < 0.05

**Supplementary Figure 4** – Proportion of patients with paravalvular leakage at 3 months, 1, 5 and 10 years of follow-up


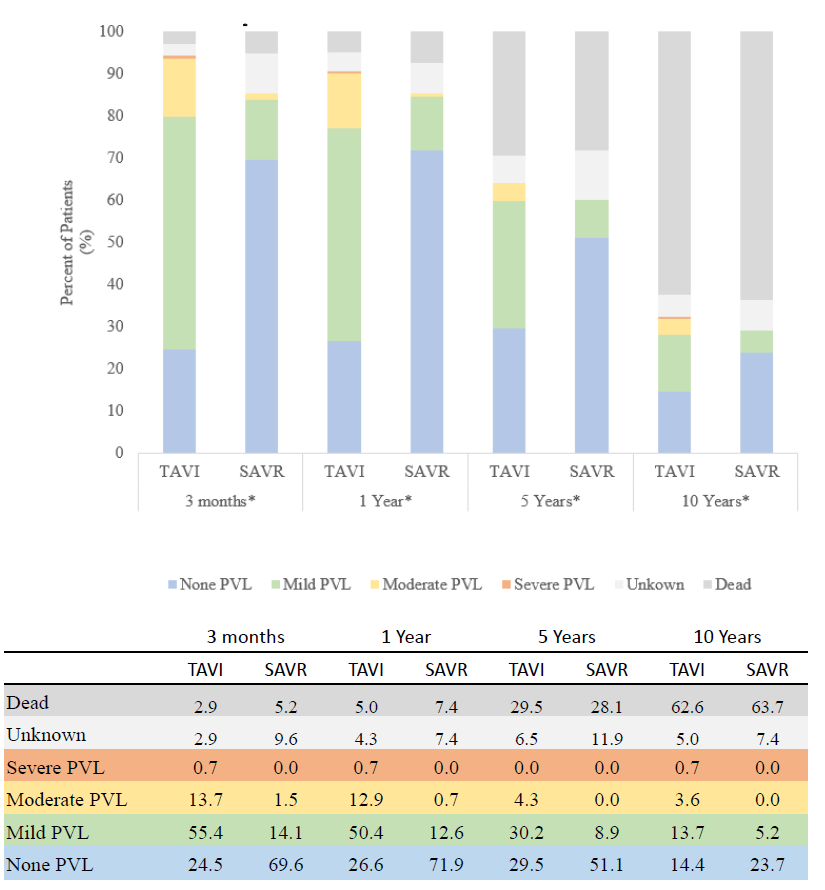


PVL: paravalvular leakage; SAVR: surgical aortic valve replacement (n = 135); TAVI: transcatheter aortic valve implantation (n = 139)

*p < 0.05 for intergroup comparison

**Supplementary Figure 5** – Proportion of patients with intraprosthetic regurgitation at 3 months, 1, 5 and 10 years of follow-up


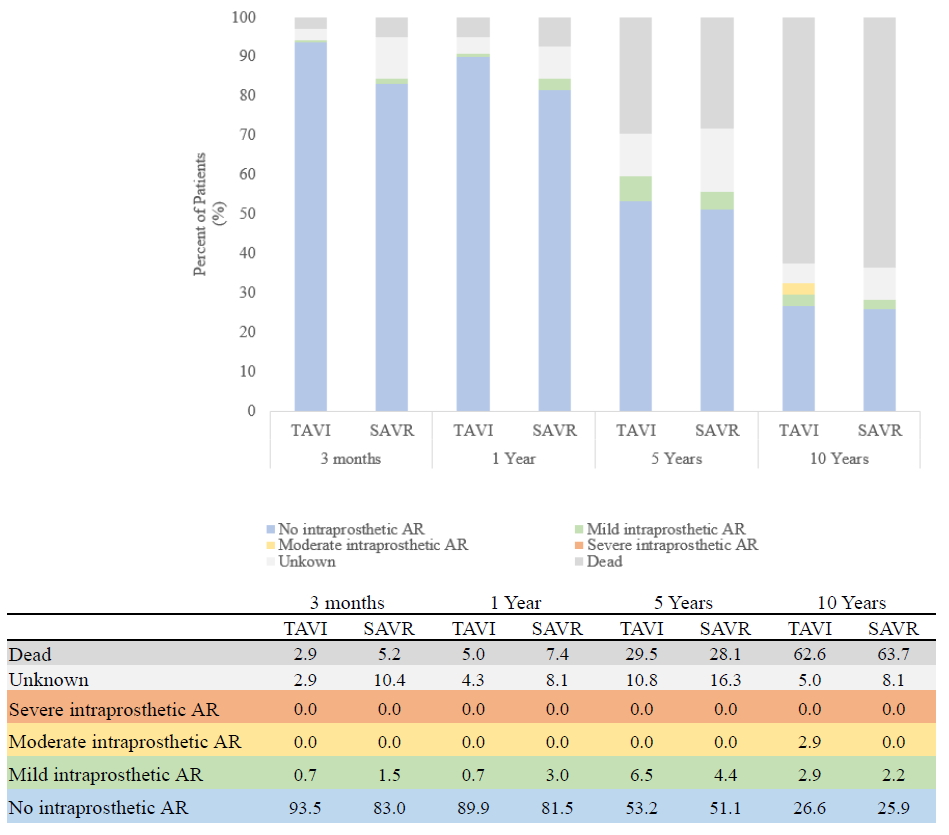


AR: aortic regurgitation; SAVR: surgical aortic valve replacement (n = 135); TAVI: transcatheter aortic valve implantation (n = 139)

*p < 0.05 for intergroup comparison

**Supplementary Figure 6** – Proportion of patients with total aortic bioprosthetic regurgitation at 3 months, 1, 5 and 10 years of follow-up


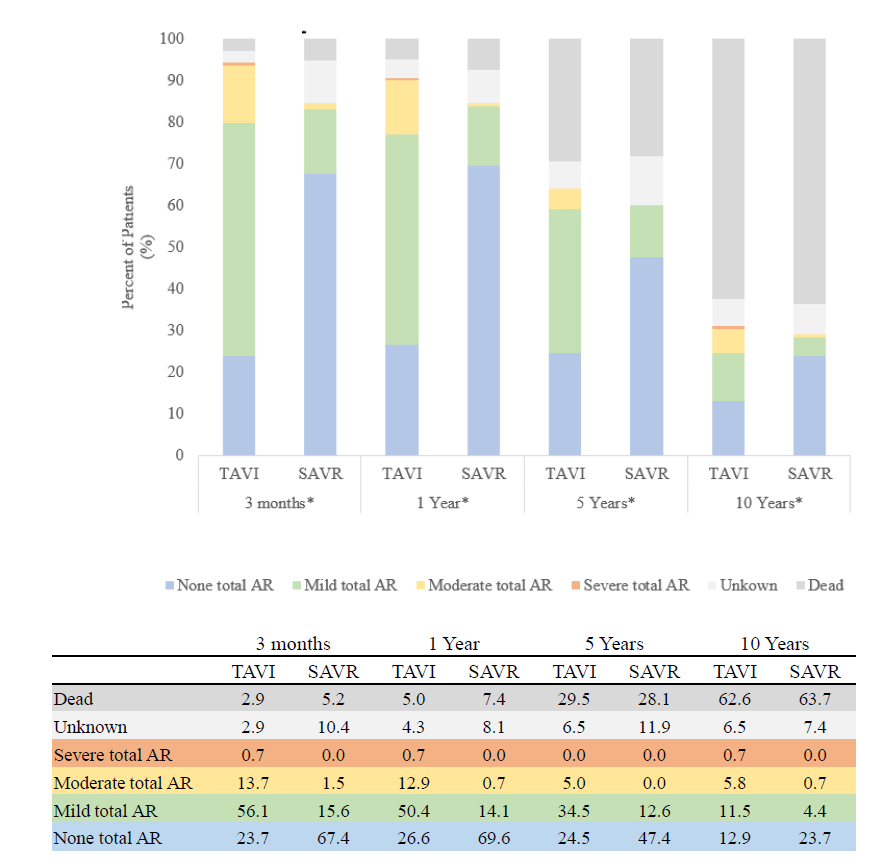


AR: aortic regurgitation; SAVR: surgical aortic valve replacement (n = 135); TAVI: transcatheter aortic valve implantation (n = 139)

*p < 0.05 for intergroup comparison

**Supplementary Figure 7** – Structural valve deterioration (SVD) defined according to VARC-3 criteria excluding Doppler velocity index (DVI)


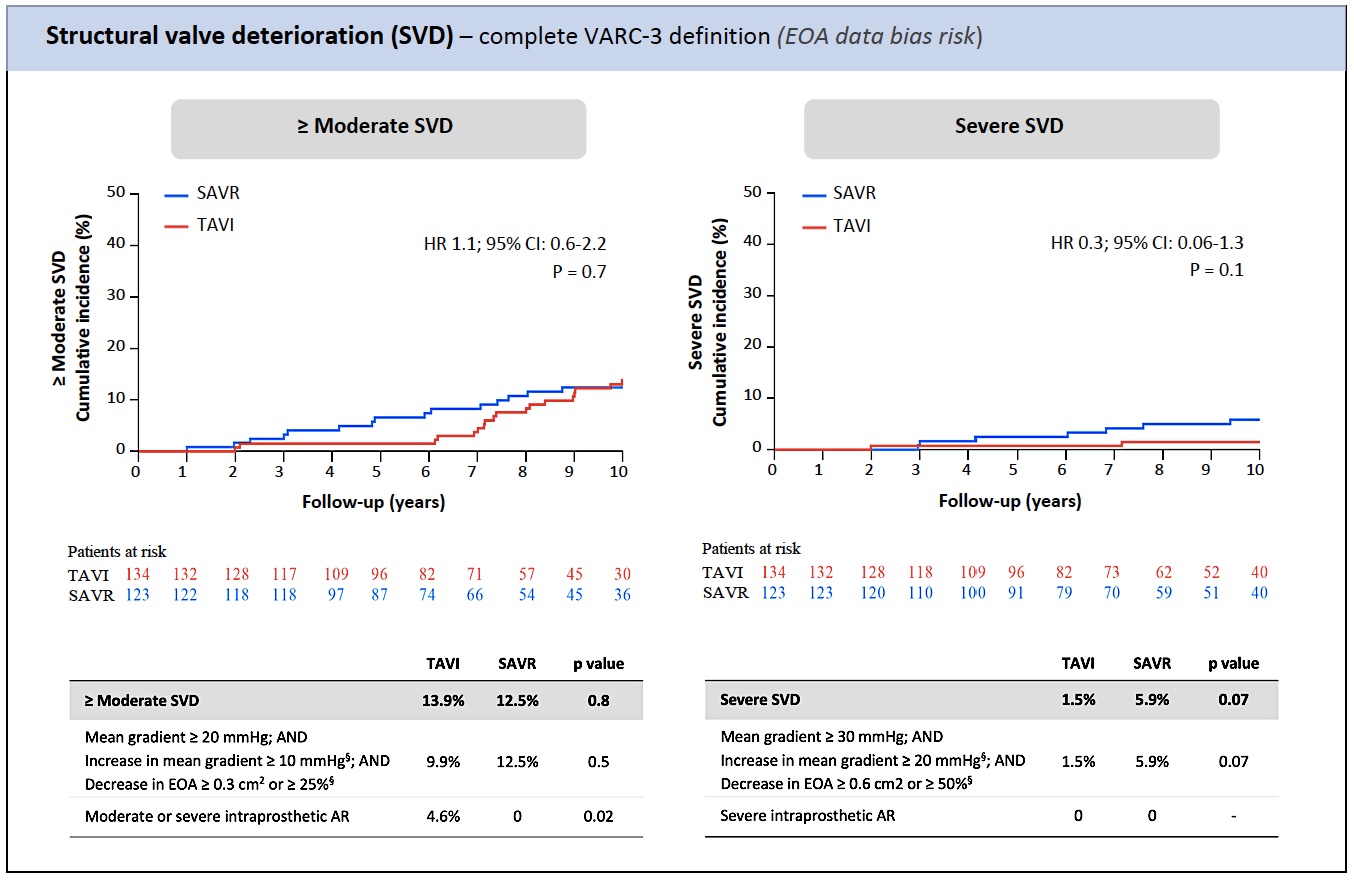


SVD, structural valve deterioration; EOA, effective orifice area; SAVR, Surgical aortic valve replacement; TAVI, Transcatheter aortic valve implantation; AR, aortic valve regurgitation.

Structural valve deterioration based on VARC-3 criteria excluding DVI.

Table and curve are cumulative incidences after 10 years of follow-up; compared with Gray’s test.

Hazard ratio (HR); 95% confidence interval (CI) and p-value in figure are based of Cox Regression.

**Supplementary Figure 8** – Bioprosthetic valve dysfunction (BVD) and failure (BVF) defined according to VARC-3 criteria excluding Doppler velocity index (DVI)


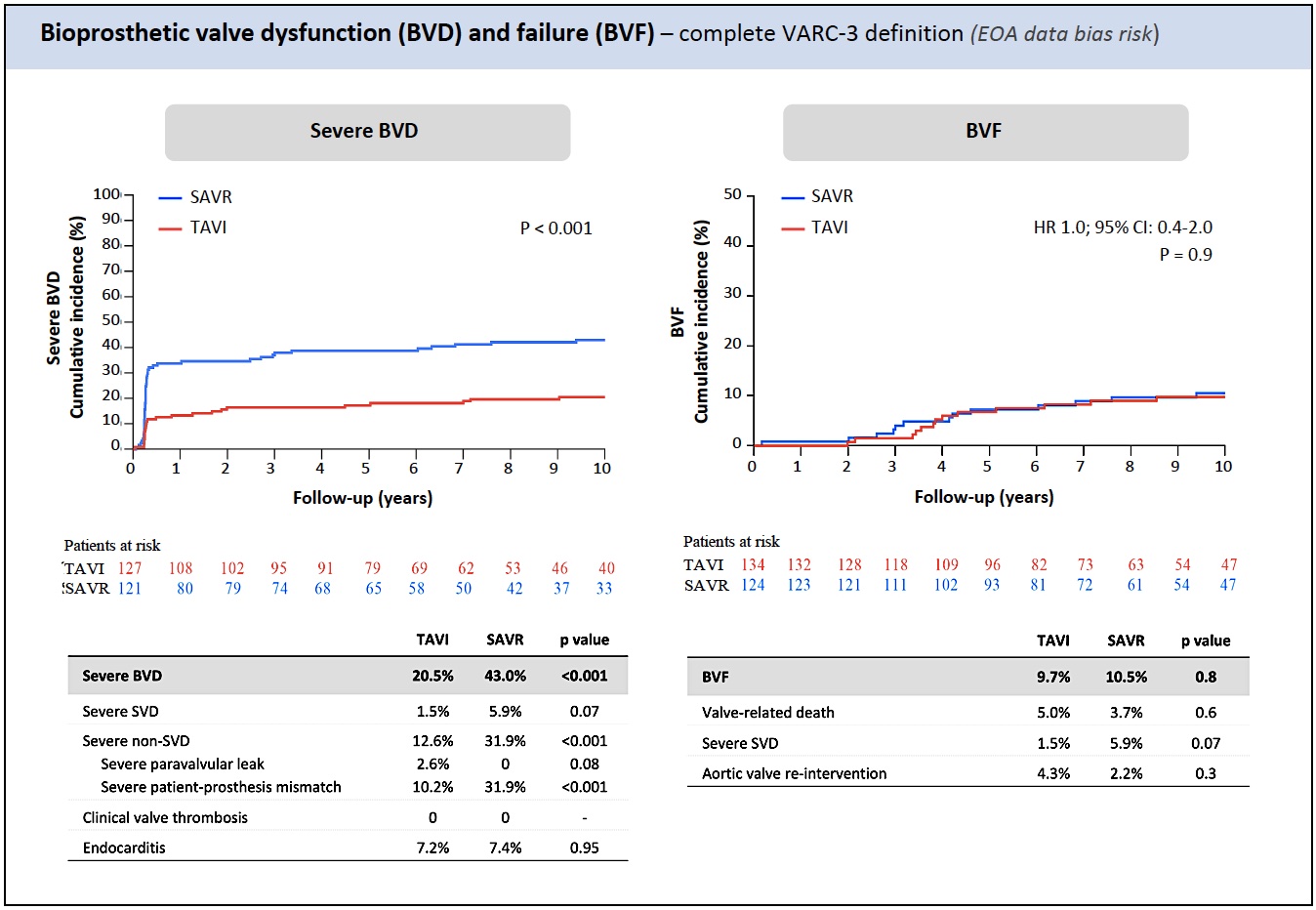


BVD: bioprosthetic valve deterioration; BVF, bioprosthetic valve failure; EOA, effectice orifice area; SVD, structural valve deterioration; SAVR, Surgical aortic valve replacement; TAVI: Transcatheter aortic valve implantation.

Structural valve deterioration based on VARC-3 criteria excluding DVI.

Table and curve are cumulative incidences after 10 years of follow-up; compared with Gray’s test.

Hazard ratio (HR); 95% confidence interval (CI) and p-value in figure are based of Cox Regression.
